# Discrimination of Pesticide Residue Levels on the Hami Melon Surface Using Multiscale Convolution

**DOI:** 10.3390/foods11233881

**Published:** 2022-12-01

**Authors:** Guowei Yu, Benxue Ma, Huihui Li, Yating Hu, Yujie Li

**Affiliations:** 1 College of Mechanical and Electrical Engineering, Shihezi University, Shihezi 832003, China; 2 Key Laboratory of Northwest Agricultural Equipment, Ministry of Agriculture and Rural Affairs, Shihezi 832003, China; 3 Analysis and Testing Center, Xinjiang Academy of Agricultural and Reclamation Sciences, Shihezi 832000, China; 4 Food Quality Supervision and Testing Center (Shihezi), Ministry of Agriculture and Rural Affairs, Shihezi 832000, China

**Keywords:** safety detection, pesticide residues, convolutional neural network, visible/near-infrared spectroscopy, Hami melon

## Abstract

Pesticide residues directly or indirectly threaten the health of humans and animals. We need a rapid and nondestructive method for the safety evaluation of fruits. In this study, the feasibility of visible/near-infrared (Vis/NIR) spectroscopy technology was explored for the discrimination of pesticide residue levels on the Hami melon surface. The one-dimensional convolutional neural network (1D-CNN) model was proposed for spectral data discrimination. We compared the effect of different convolutional architectures on the model performance, including single-depth, symmetric, and asymmetric multiscale convolution. The results showed that the 1D-CNN model could discriminate the presence or absence of pesticide residues with a high accuracy above 99.00%. The multiscale convolution could significantly improve the model accuracy while reducing the modeling time. In particular, the asymmetric convolution had a better comprehensive performance. For two-level discrimination, the accuracy of lambda-cyhalothrin and beta-cypermethrin was 93.68% and 95.79%, respectively. For three-level discrimination, the accuracy of lambda-cyhalothrin and beta-cypermethrin was 86.32% and 89.47%, respectively. For four-level discrimination, the accuracy of lambda-cyhalothrin and beta-cypermethrin was 87.37% and 93.68%, respectively, and the average modeling time was 3.5 s. This finding will encourage more relevant research to use multiscale 1D-CNN as a spectral analysis strategy for the detection of pesticide residues in fruits.

## 1. Introduction

Hami melon is one of the famous and special products in Xinjiang, tasting delicious and enjoying the reputation of “the king of melons” [1]. The safety of fruits and vegetables has always been the focus of society. In recent years, the problem of pesticide residues in Hami melon has become more and more serious [2]. Pyrethroid pesticides are often used for pest control during Hami melon planting [3]. The residual pesticides attach to the surface of Hami melon and continue to contaminate the fruit. The rind of the Hami melon is often fed to the livestock [4], and could also be prepared as dietary fiber [5]. In addition, Hami melon is a fresh-eating food, and we consume its pulp. When we cut the Hami melon, the knife touches the surface with pesticide residues, which could contaminate the pulp and pose a potential food safety risk. Pesticide residues not only cause food safety problems, but also directly or indirectly threaten the health of humans and animals. Therefore, it is urgent to achieve rapid discrimination of pesticide residue levels on the Hami melon surface to ensure its quality and safety in the market [6].

Conventional chemical methods for the detection of pesticide residues in fruits and vegetables mainly include gas chromatography (GC), high-performance liquid chromatography (HPLC), gas/liquid chromatography–mass spectrometry (GC/LC-MC), and so forth [7]. These detection methods have high accuracy and sensitivity, but the detection steps are complex and costly [8]. As a rapid modern detection technique without sample pretreatment, visible/near-infrared (Vis/NIR) spectroscopy has been gradually applied in the quality and safety detection of fruits and vegetables [9,10,11], especially in qualitative discrimination, including type and level. Sun et al. [12] established an optimized support vector machine (SVM) model using near-infrared transmission spectroscopy (950–1650 nm), which could identify two pesticide residue types (fenvalerate and chlorpyrifos) in lettuce leaves, and the prediction accuracy was 98.33%. Zhou et al. [13] used Vis/NIR polarization spectroscopy (300–1000 nm) to identify five pesticide residue types (avermectin, dichlorvos, dimethoate, phoxim, and acephate) in lettuce leaves, achieving a prediction accuracy of 97.78%. Ndung’u et al. [14] used principal component analysis (PCA) to reduce the dimensionality of Vis/NIR spectra (325–1075 nm), and established a machine learning model to identify pesticide residues (mixtures of beta-cyfluthrin and chlorpyrifos, mixtures of metalaxyl and mancozeb) in spinach. The model obtained a perfect prediction accuracy of 100.00%. Li et al. [15] proposed an all-band average grouping integration preprocessing method based on Vis/NIR spectra (350–2500 nm), which could realize the four-level discrimination of chlorpyrifos residues in cabbage leaves. This method outperformed spectral-sensitive band selection, and achieved a higher prediction accuracy of 96.67%. Nazarloo et al. [16] demonstrated the feasibility of using Vis/NIR spectroscopy (400–1050 nm) and multivariate analysis for the two-level discrimination of profenofos residues in tomatoes, and the prediction accuracy was 91.66%. Recent studies mainly focus on the identification of the presence or absence of pesticide residues and the residue types. There are few studies on the discrimination of pesticide residue levels in fruits.

Generally, model accuracy can be improved by combining various methods, such as preprocessing, feature selection, and modeling. However, it could increase the model complexity, and modeling time [17]. It is always a great challenge to extract and use Vis/NIR spectral features effectively. Deep neural networks can automatically learn critical patterns from massive raw data by end-to-end analysis, which reduces the need for feature engineering [18]. Recent developments in spectral analysis have demonstrated that deep learning combined with spectroscopic sensing techniques for the quality and safety evaluation of agro-products increases attention [19], and the deep learning algorithm has shown great potential for pesticide residue discrimination of fruits and vegetables. The deep brief network (DBN) was used to select and extract spectral features, achieving the identification of fenvalerate and triazoline residues in lettuce leaves [20]. The residual neural network (ResNet) was shown to have a good effect on three-level residue discrimination in grapes [21]. Moreover, the one-dimensional convolutional neural network (1D-CNN) achieved the identification of pesticide residues on garlic chive leaves (λ-cyhalothrin, trichlorfon, phoxim, mixtures of trichlorfon and phoxim) [22], and also worked well on Hami melon (chlorothalonil, imidacloprid, and pyraclostrobin) [23]. To the best of our knowledge, the use of multiscale convolutional architecture for the discrimination of pesticide residue levels has not been investigated yet.

The objectives of this study were (1) to explore the feasibility of Vis/NIR spectroscopy combined with 1D-CNN models for the discrimination of pesticide residue levels on the Hami melon surface; (2) to evaluate the impact of multiscale convolutional architecture on model performance; and (3) to explore the effect of increasing complexity (more levels) on model performance for the discrimination of pesticide residues.

## 2. Materials and Methods

### 2.1. Sample Preparation

**Figure 1:**
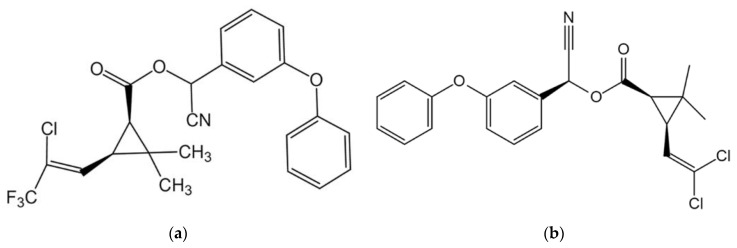
The chemical molecular structures of pesticides. (**a**) Lambda-cyhalothrin; (**b**) Beta-cypermethrin.

A total of 140 Hami melons (variety: Xizhoumi No. 25), with a weight of (2.8 ± 0.4) kg, were obtained from a local agricultural product trading center in Shihezi, Xinjiang, China. The lambda-cyhalothrin (2.5%, microemulsion, Shandong Caoda Chemical Co., Ltd., Heze China) and beta-cypermethrin (4.5%, emulsifiable concentrate, Jinan Yinong Chemical Co., Ltd., Jinan, China), as pesticides commonly used during Hami melon planting, were obtained from a local agricultural material market in Shihezi, Xinjiang, China. Figure 1 shows the chemical molecular structures of two pesticides.

In order to reduce the impact of environmental factors on this experiment, Hami melons were wiped clean and then placed in a laboratory with constant temperature (25 °C) and relative humidity (30%) for 24 h. A total of 140 Hami melons were divided into four groups. Compound pesticide solutions of beta-cyhalothrin (A), beta-cypermethrin (B), and water (C) were prepared with a ratio of 1:200, 1:400, and 1:800. Three groups of Hami melons were evenly sprayed with compound pesticide solutions. The remaining 35 Hami melons were used as a control group sprayed with clean water. The treated samples were allowed to dry and ventilated at the same temperature and relative humidity for 10 h.

### 2.2. Spectral Data Acquisition

**Figure 2:**
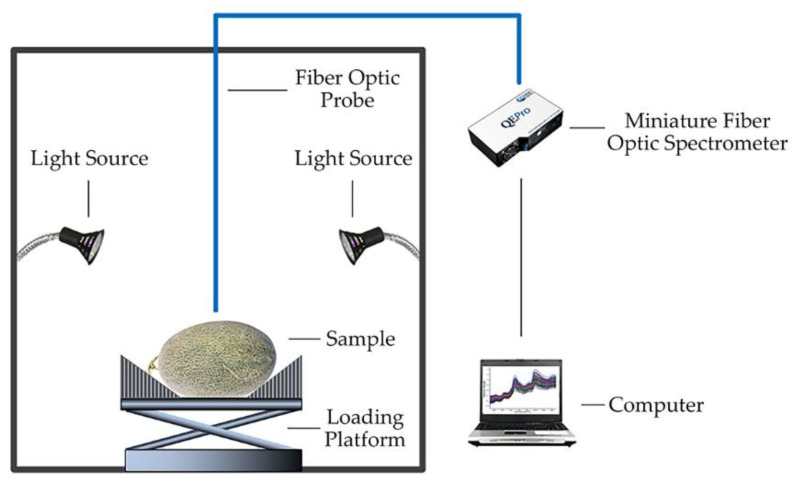
Vis/NIR spectral data acquisition system. Note: Cited from Yu et al. [23].

The spectra of pesticide residues on the Hami melon surface were collected by a Vis/NIR (380–1100 nm) spectral data acquisition system, which consisted of a miniature fiber optic spectrograph with a spectral resolution of 0.69 nm (QE Pro-FL, Ocean Insight, Inc., Dunedin, FL, USA), a fiber optic probe (QP600-2-VIS-NIROOS-00-5172-11, Ocean Insight, Inc., Dunedin, FL, USA), an illumination unit consisting of two halogen light sources (MR16, Signify(China) Investment Co., Ltd., Shanghai, China), a loading platform and a computer installed with spectrometer operating software (OceanView v1.6.7, Ocean Insight, Inc., Dunedin, FL, USA), as shown in Figure 2. The distance from the optical fiber probe to the Hami melon surface was about 3 cm. Before acquiring the spectra, the integration time was set at 100 ms, and the moving average width and average number of scans were set at 4 and 10, respectively. In this experiment, the original spectra were collected at the equator position, and the interval angle between each sampling point was 90°.

### 2.3. Pesticide Residue Content Measurement

After the spectral data acquisition, all Hami melons were sent to the Food Quality Supervision and Testing Center (Shihezi), Ministry of Agriculture and Rural Affairs. The method of GC combined with QuEChERS (acronym of quick, easy, cheap, effective, rugged, and safe) was followed to measure pesticide residues (lambda-cyhalothrin and beta-cypermethrin) in Hami melon, according to Chinese Standard (NY/T 761-2008) and British Standard (BS EN 15662: 2008) [24,25]. There were five steps to measure pesticide residue contents.

(1) Standard preparation

The certified pesticide standard solution, with a concentration of 1000 mg/L and purities greater than 98.0%, was purchased from the Agro-Environmental Quality Supervision and Testing Center, Ministry of Agriculture and Rural Affairs (Tianjin, China). Standard mixture intermediate and working solutions were prepared in n-hexane (chromatographically pure) (CAS 110-54-3, Duksan Pure Chemicals Co., Ltd., Ansan-si, Korea) at a concentration of 20.0 mg/mL and 1.0 mg/mL, respectively. The solutions were stored in brown reagent bottles at 4 °C, and placed at room temperature before use.

(2) Sample Preparation

The pulps and rinds of each Hami melon were cut into samples with a thickness of about 1.50 cm. And samples were crushed in a food processor. Then the treated samples were transferred to the marked sample bottles. They were stored at −18 °C, and placed at room temperature before measurement.

(3) Extraction

A 7.5 g amount of the crushed sample was weighed by an electronic balance (BSA4202S-CW, Sartorius Inc., Gottingen, Germany), and was transferred to a 50 mL centrifuge tube. Then, 15 mL of acetonitrile (chromatographically pure, ANPEL Scientific Instrument (Shanghai) Co., Ltd., China) was added. The mixture was vortexed at a speed of 3000 r/min by a vortex shaker (MS 3 Control, IKA Inc., Staufen, Germany) for 40 s. After homogenization for 1 min, 5 g of NaCl was added to the mixture and again vortexed at a speed of 3000 r/min for 40 s. Subsequently, the tubes were centrifuged by a high-speed centrifuge (TG16-WS, Xiangyi Centrifuge Instrument Co., Ltd., Changsha, China) at a speed of 7000 r/min for 5 min to separate the two layers. An 8 mL volume of the supernatant was removed for clean-up.

(4) Clean-up

An 8 mL volume of the supernatant was transferred to a 15 mL QuEChERS clean-up centrifuge tube (5982-0029, Agilent Technologies Inc., Santa Clara, CA, USA), which contained 400.1 mg PSA, 400.1 mg C18 EC, 45.0 mg bulk carbograph, and 1199.8 mg magnesium sulfate (purity from 98.5% to 101.5%). Then, the mixture was vortexed at a speed of 3000 r/min for 40 s and centrifuged at a speed of 7000 r/min for 5 min. A 4 mL volume of the supernatant was transferred to a glass tube and evaporated to dryness by a nitrogen evaporator (N-EVAP-112, Organomation Associates, Inc., Burlington, VT, USA). Finally, the extract was redissolved in 2 mL of n-hexane and measured by GC.

(5) GC conditions

The gas chromatograph (Agilent 7890A, Agilent Technologies Inc., Santa Clara, CA, USA) was equipped with a micro electron capture detector (μ-ECD). Separation of the pesticides was achieved on a fused silica capillary tubing column (HP-5, Agilent Technologies Inc., Santa Clara, CA, USA) with a size of 30 m × 0.320 mm × 0.25 μm (length inner diameter film thickness). Nitrogen gas (purity of about 99.999%) was used as a carrier gas at a flow rate of 2.0 mL/min. The temperatures of the inlet and detector were 220 °C and 320 °C, respectively. The flow rates of septum purge and makeup were 3.0 mL/min and 60 mL/min, respectively. The oven temperature was kept at 100 °C for 1 min, then increased to 190 °C at a rate of 15 °C/min and held for 2 min, and finally increased to 280 °C at a rate of 6 °C/min and held for 2 min. The injection volume was 1μL in splitless mode. The pesticide residue content was obtained by calculating the peak area ratio between the sample and the standard solution.

### 2.4. 1D-CNN Model Implementation and Evaluation

#### 2.4.1. Environment

The computations were performed on a Lenovo computer with a Windows 10 (64-bit) operating system, an Intel (R) Core (TM) I7-8700 @3.20 GHz CPU, an NVIDIA GeForce RTX2060 graphics card with 16.0 GB of RAM. For GPU acceleration, a computing platform (NVIDIA CUDA Toolkit 10.1) and a deep neural network acceleration library (NVIDIA cuDNN v7.6.5) were used. All models were implemented on TensorFlow 2.1.0 framework and deep learning library Keras 2.3.1 using Python 3.7.3 in Spyder IDE (v. 3.3.3).

#### 2.4.2. Architecture

Generally, the 1D-CNN model architecture has the convolution layer (labeled as Conv), the pooling layer (labeled as Pooling), the flatten layer (labeled as F), the fully connected layer (labeled as FC), and the input and output layer. In order to improve the high-level feature extraction capability, we often increase multiple processing layers. As the network depth increases, the model accuracy could be improved, but it will face the challenge of increasing the computational complexity. An Inception architecture was proposed to have width and depth while keeping the complexity constant [26]. To further optimize the network, improved Inception architectures were proposed to capture various features while balancing the width and depth [27]. In this study, we designed three 1D-CNN models to analyze spectral data, including two multiscale networks based on Inception architecture and a single-depth network. The input was the 1D spectral preprocessing data, and the output was the object class (pesticide residue levels) to be discriminated.

**Figure 3:**
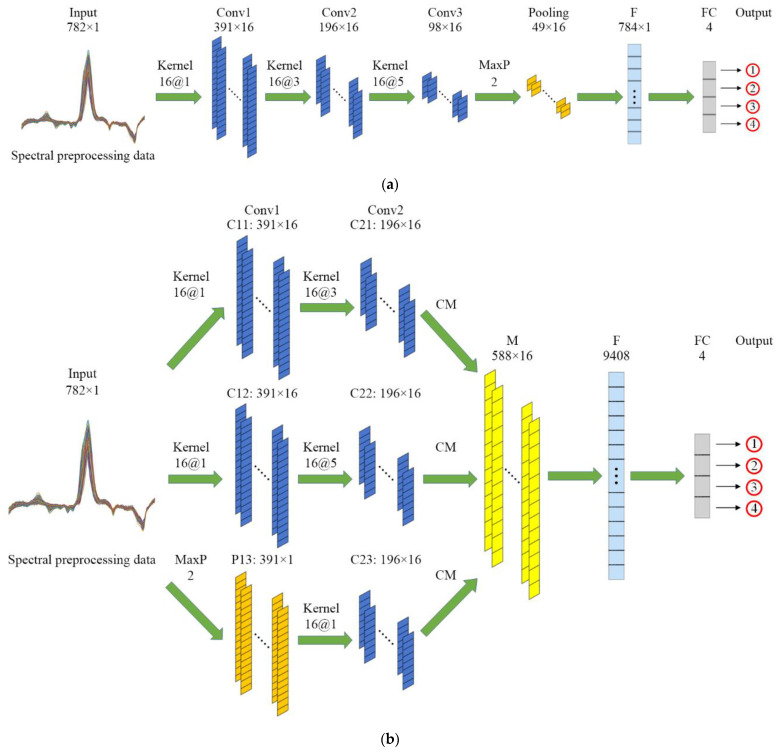

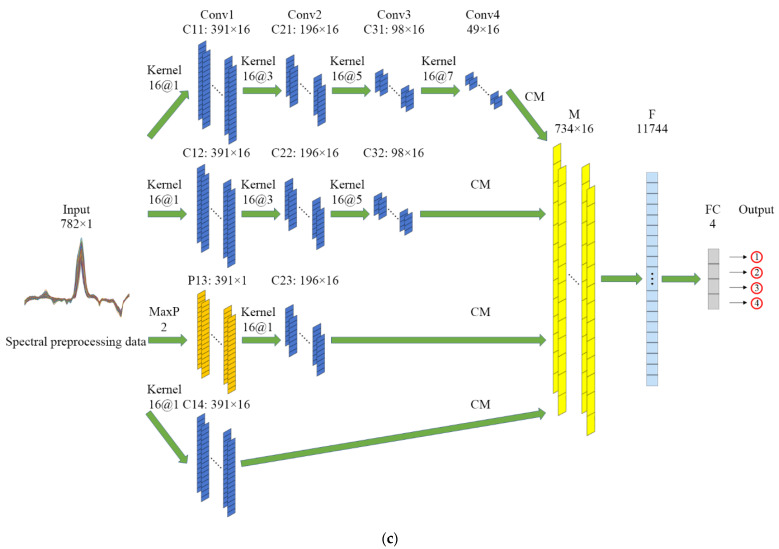
1D-CNN model architecture. (**a**) Single-depth convolution; (**b**) Symmetric multiscale convolution; (**c**) Asymmetric multiscale convolution. Here, 782 × 1 represents the model input as 1D spectral vector with a length of 782; Kernel 16@1 represents the convolution operation, the number of convolution filters is 16, the length of the 1D convolution window is 1; MaxP 2 represents the max pooling operation, the size of the max pooling window is 2; l × d represents the dimensionality of the feature vector as a network layer output, the length and depth of the feature vector are l and d, respectively; n represents the number of neurons in layer FC.

The single-depth 1D-CNN architecture included three convolution layers, a pooling layer, a flatten layer, and a fully connected layer, as shown in Figure 3a. The symmetrical multiscale 1D-CNN architecture included two convolution layers with five convolution modules (labeled as C) and a pooling module (labeled as P), a merging layer (labeled as M) with concatenate merging (labeled as CM), a flatten layer, and a fully connected layer, as shown in Figure 3b. The asymmetric multiscale 1D-CNN architecture included four convolution layers with eight convolution modules and a pooling module, a merging layer with concatenate merging, a flatten layer, and a fully connected layer, as shown in Figure 3c. The concatenate merging layer achieved multiscale feature fusion by the concatenation of the feature vectors. The fusion feature length was the sum of the feature vector lengths extracted by the parallel convolution modules, and the width and depth were kept constant [28].

#### 2.4.3. Hyperparameters

Table 1 shows the hyperparameters used in the different network layers in three 1D-CNN models. The 1 × 1 convolution kernel was used for reducing the data dimensions. The features of different scales were captured by n × 1 convolution kernels. The padding strategy was selected to keep the size of the feature map. According to previous studies [21,22,23], the activation, objective, and loss functions were commonly used as rectified linear unit (ReLU), softmax, and multiclassification cross-entropy, respectively. The stochastic gradient descent (SGD) optimizer, with a momentum of 0.6 and decay of 1 × 10^−5^, was used to improve the training process. According to the exponential scale ($10^{-n}$ and $2^{n}$) [29], the learning rate and batch size were selected as 0.01 and 64, respectively. Dropout with a rate of 0.2 was adopted to reduce the number of parameters and prevent overfitting.

#### 2.4.4. Evaluation

The model performance was evaluated using a normalized confusion matrix based on the test dataset [30]. Table 2 shows an illustration of normalized confusion matrix in two-class discrimination.

The accuracy, true positive rate (*TPR*), true negative rate (*TNR*), false negative rate (*FNR*) and false positive rate (*FPR*) are calculated in Equations (1)–(5).

(1) $$Accuracy=\frac{TP+TN}{TP+FN+TN+FP}$$

(2) $$TPR=\frac{TP}{TP+FN}$$

(3) $$TNR=\frac{TN}{TN+FP}$$

(4) $$FNR=\frac{FN}{TP+FN}$$

(5) $$FPR=\frac{FP}{TN+FP}$$

where TP is true positive (samples with the actual positive label were predicted to be positive); TN is true negative (samples with the actual negative label were predicted to be negative); FN is false negative (samples with the actual positive label were predicted to be negative); FP is false positive (samples with the actual negative label were predicted to be positive).

## 3. Results and Discussion

### 3.1. Data Statistics and Division

Due to individual differences, the measurement contents of pesticide residues in five Hami melons were abnormal, and their corresponding 20 spectra were not used. In addition, 20 abnormal spectra were manually removed. Thus, a total of 520 spectral data were used in this study, including 380 samples with pesticide residues and 140 samples without pesticide residues. In order to evaluate the 1D-CNN model performance with the level increases, we removed outliers and made spectral data as uniformly distributed as possible. Table 3, Table 4 and Table 5 show the spectral data distribution in two-, three- and four-level discrimination of pesticide residues, respectively. The measurement results showed that the residual content of lambda-cyhalothrin on the Hami melon surface was higher than that of beta-cypermethrin. The max and min residual contents of lambda-cyhalothrin were 32.36 and 0.96 μg/mL, respectively. The max and min residual contents of beta-cypermethrin were 12.74 and 0.37 μg/mL, respectively.

In order to eliminate noise and baseline shift in the raw spectra, the first-order derivative computation using the Savitzky–Golay algorithm was used to preprocess the spectral data [31]. The number of points in the filter and the order of the polynomial were five and two, respectively. The spectral data were then divided into training and test sets in a 3:1 ratio.

### 3.2. Interpretation of Vis/NIR Spectra

**Figure 4:**
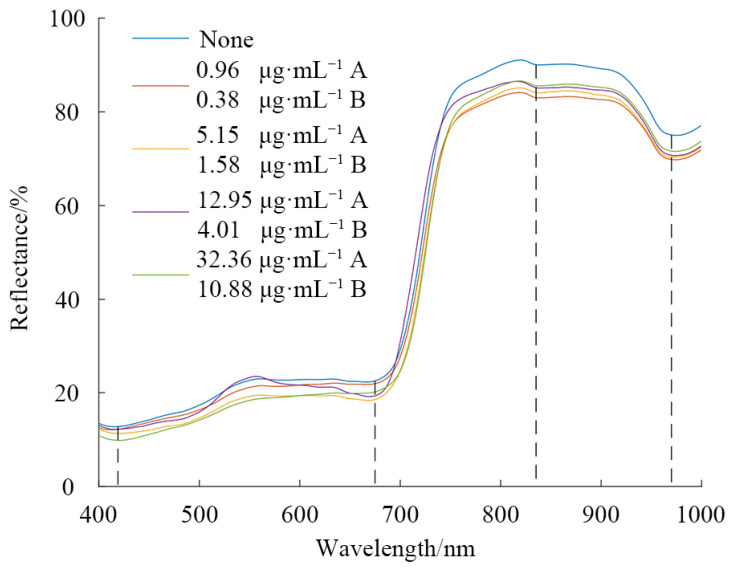
Vis/NIR spectra of different compound pesticide residue content on the Hami melon surface. A is lambda-cyhalothrin; B is beta-cypermethrin.

Figure 3 shows the Vis/NIR spectral average reflectance of pesticide residues on the Hami melon surface. The pesticide residue content shown in Figure 4 was min, three-quantile, and max, respectively. It can be seen that the overall trends of the spectra were almost similar. The spectral reflectance of the Hami melon without pesticide residues was visibly higher after 760 nm. As can be observed, there were two slight absorbance peaks around 420 and 675 nm, which were possibly related to the carotenoids and chlorophylls [32]. The surface colors of the Hami melon were caused by a combination of pigments, the most visible of carotenoids and chlorophylls. The spectral reflectance showed a rapidly increasing trend at 690–760 nm due to the “red edge” of the plant [33]. The weak absorbance peak at approximately 835 nm was associated with the third overtone of the C-H functional group [34]. The obvious absorbance peak around 980 nm was closely related to the second overtone of the O-H group [35]. This was attributed to the moisture change in the Hami melon. The pesticide residues did not change the position of the spectral feature absorbance peaks, and it agreed with previous studies such as Ye et al. [21], Yu et al. [23], and Sun et al. [36]. The spectral curves of different pesticide residue contents in different mature periods overlapped partially, and the difference was not obvious. Therefore, the pesticide residue levels on the Hami melon surface cannot be directly distinguished by the raw Vis/NIR spectral reflectance. It is necessary to carry out further spectral analysis through the deep learning approach.

### 3.3. 1D-CNN Model

#### 3.3.1. Pesticide Residue Discrimination

**Figure 5:**
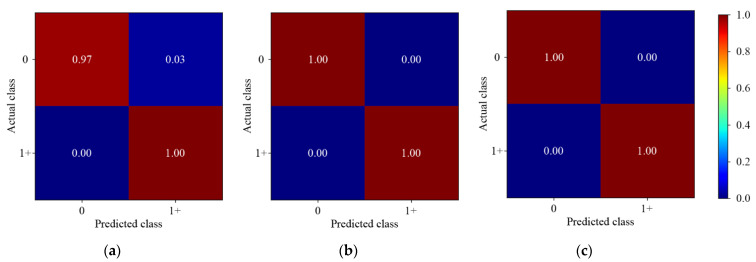
The normalized confusion matrix for pesticide residue discrimination. (**a**) Single-depth 1D-CNN; (**b**) Symmetric multiscale 1D-CNN; (**c**) Asymmetric multiscale 1D-CNN. Label 0 represents samples without pesticide residues; Label 1+ represents samples with pesticide residues.

Figure 5 shows the results of different 1D-CNN models for pesticide residue discrimination. Three 1D-CNN models could accurately discriminate the samples with pesticide residues. However, the prediction of the single-depth 1D-CNN model was not perfect, 3.00% of the samples without pesticide residues were miscategorized. The results indicated that 1D-CNN models could discriminate the presence or absence of pesticide residues on the Hami melon surface. Compared with the single-depth convolution, the multiscale convolution had a slight advantage.

#### 3.3.2. Two-Level Residue Discrimination

**Figure 6:**
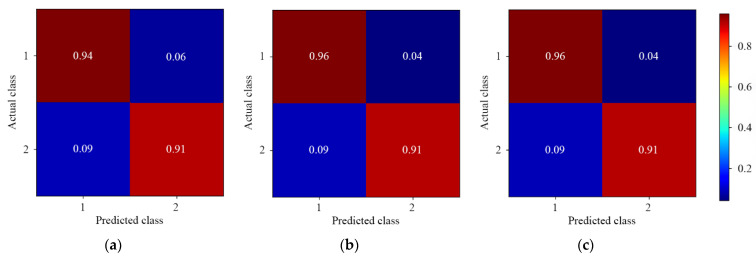
The normalized confusion matrix for two-level discrimination of lambda-cyhalothrin residues. (**a**) Single-depth 1D-CNN; (**b**) Symmetric multiscale 1D-CNN; (**c**) Asymmetric multiscale 1D-CNN.

**Figure 7:**
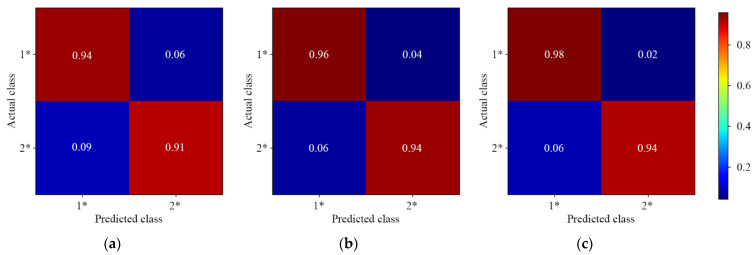
The normalized confusion matrix for two-level discrimination of beta-cypermethrin residues. (**a**) Single-depth 1D-CNN; (**b**) Symmetric multiscale 1D-CNN; (**c**) Asymmetric multiscale 1D-CNN.

Figure 6 and Figure 7 show the results of different 1D-CNN models for two-level discrimination of lambda-cyhalothrin and beta-cypermethrin residues, respectively. The prediction for the three models was similar at high accuracy. In particular, the residue concentration of below 8.50 μg/mL labeled as 1 and 1* was predicted quite well. This was probably due to the spectral depth feature of the two-level pesticide residues being obvious. Moreover, the two-level residue discrimination of beta-cypermethrin was better than that of lambda-cyhalothrin. It indicated that 1D-CNN models might be more appropriate for the detection of beta-cypermethrin residues. For model performance, two multiscale 1D-CNN models outperformed the single-depth model. For the asymmetric multiscale 1D-CNN model, the accuracy for low-level (labeled as 1 and 1*) residues was 96.00% and 98.00%, respectively.

#### 3.3.3. Three-Level Residue Discrimination

**Figure 8:**
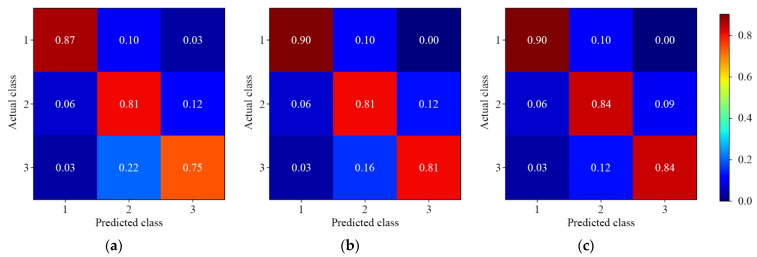
The normalized confusion matrix for three-level discrimination of lambda-cyhalothrin residues. (**a**) Single-depth 1D-CNN; (**b**) Symmetric multiscale 1D-CNN; (**c**) Asymmetric multiscale 1D-CNN.

**Figure 9:**
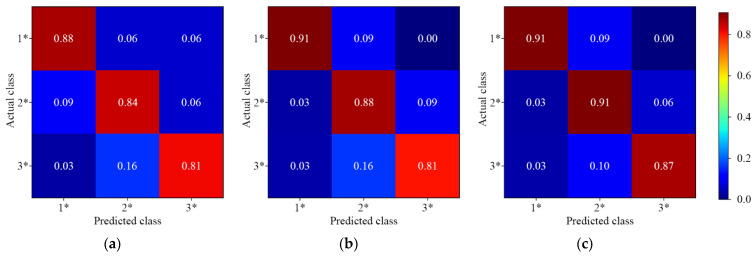
The normalized confusion matrix for three-level discrimination of beta-cypermethrin residues. (**a**) Single-depth 1D-CNN; (**b**) Symmetric multiscale 1D-CNN; (**c**) Asymmetric multiscale 1D-CNN.

Figure 8 and Figure 9 show the results of different 1D-CNN models for three-level discrimination of lambda-cyhalothrin and beta-cypermethrin residues, respectively. It can be found that the overall prediction accuracy was reduced. The actual medium level (labeled as 2 and 2*) was predicted into low and high levels. This phenomenon could be caused by the similarity of the spectral features. The asymmetric multiscale convolution was able to improve the model accuracy. Almost none of the low level was predicted as high level, and only 3.00% of the high level was predicted as low level. However, the accuracy of lambda-cyhalothrin residues was still below 85.00% for the discrimination of medium and high level. The results showed that the three-level residue discrimination of beta-cypermethrin was better than that of lambda-cyhalothrin.

#### 3.3.4. Four-Level Residue Discrimination

**Figure 10:**
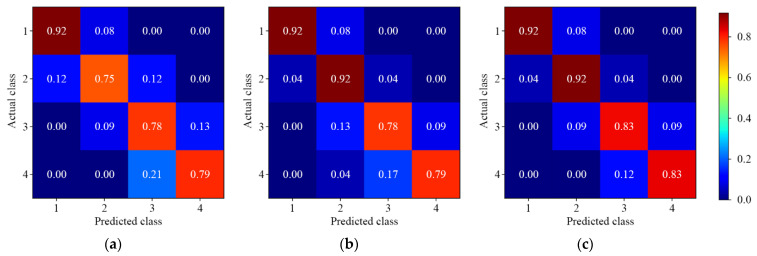
The normalized confusion matrix for four-level discrimination of lambda-cyhalothrin residues. (**a**) Single-depth 1D-CNN; (**b**) Symmetric multiscale 1D-CNN; (**c**) Asymmetric multiscale 1D-CNN.

**Figure 11:**
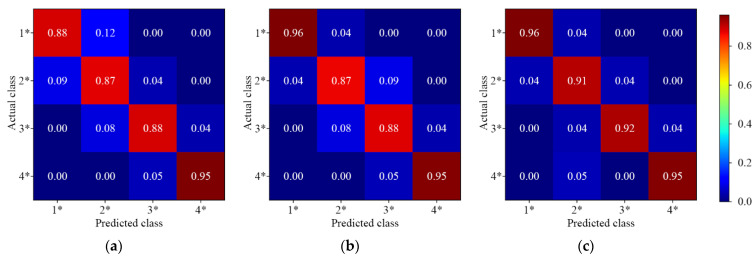
The normalized confusion matrix for four-level discrimination of beta-cypermethrin residues. (**a**) Single-depth 1D-CNN; (**b**) Symmetric multiscale 1D-CNN; (**c**) Asymmetric multiscale 1D-CNN.

Figure 10 and Figure 11 show the results of different 1D-CNN models for four-level discrimination of lambda-cyhalothrin and beta-cypermethrin residues, respectively. For the discrimination of lambda-cyhalothrin residues, the misclassification was serious in Levels 3 and 4, which was similar to three-level discrimination results. The overall results showed that the model could perfectly differentiate the low (labeled as 1 and 1*) and high (labeled as 4 and 4*) levels a misclassification of 0%. The discrimination of beta-cypermethrin residues was better with an accuracy of more than 90.00%. The four-level discrimination of beta-cypermethrin was better than that of lambda-cyhalothrin, which was consistent with two- and three-level discrimination.

#### 3.3.5. Comprehensive Evaluation

Table 6 shows the overall test results of 1D-CNN models for the discrimination of pesticide residue levels on the Hami melon surface. For the discrimination of the presence or absence of pesticide residues, the multiscale 1D-CNN models achieved better accuracy of 100.00%. Based on NIR spectroscopy, Xie et al. [37] proposed the SVM model with Hodrick–Prescott decomposition, achieving an accuracy of 71.18%. Chen et al. [38] achieved a good result that the chlorpyrifos residues could be clearly discriminated in apples and pears. In contrast, the 1D-CNN models proposed in this study had better discrimination ability.

For two-level discrimination, the accuracy of the three models was higher than 92.00%. Jamshidi et al. [39,40] and Nazarloo et al. [16] used Vis/NIR spectroscopy combined with partial least-squares discriminant analysis (PLS-DA) to discriminate pesticide residues (safe and unsafe) on cucumber and tomato surfaces. The training accuracy of the PLS-DA model was less than 100.00%, which was inferior to the multiscale 1D-CNN model, and its test accuracy of 92.31% and 91.66% was slightly lower than that of 1D-CNN. This showed that the model performance of 1D-CNN was better than PLS-DA without the feature selection. For three-level discrimination of lambda-cyhalothrin and beta-cypermethrin residues, the accuracy of the asymmetric multiscale 1D-CNN model was 86.32% and 89.47%, respectively. This result was obviously better than other models. However, it was lower in comparison to Ren et al. [41], who used the chisquare test combined with linear discriminant analysis, and Ye et al. [21], who used ResNet or logistic regression. However, they did not measure the actual residue contents, and divided residue levels according to the ratio of pesticides to water. Thus, our experimental design was more reasonable. For four-level discrimination, the 1D-CNN model accuracy was improved. Sun et al. [36] proposed a method based on piecewise discrete wavelet transform and SVM, achieving an accuracy of 90.63% for the discrimination of dimethoate residues on lettuce leaves. Compared with their findings, the discrimination capability of multiscale 1D-CNN models was weaker for lambda-cyhalothrin residues and stronger for beta-cypermethrin residues. The results demonstrated that the choice of a suitable spectral feature selection method may strongly affect the analysis performance of the conventional modeling methods. The 1D-CNN model reduced the need for human effort in feature selection.

As can be seen, the multiscale convolution provided a higher accuracy and a lower modeling time. The 1D-CNN model architecture increased the width and depth by stacking parallel n × 1 convolutions, which can improve the model accuracy while reducing the computational complexity. It was consistent with the findings of Zhang et al. [42]. The overall results showed that the asymmetric multiscale 1D-CNN model provided better performance. This could be due to the asymmetric convolution module having a stronger capability to capture various spectral features.

## 4. Conclusions

This study presented 1D-CNN models of Vis/NIR spectral analysis for rapid discrimination of compound pesticide residues on the Hami melon surface. The 1D-CNN model could discriminate the presence or absence of pesticide residues with high accuracy. For two-level residue discrimination, the model results were acceptable with an accuracy of above 92.00%. However, the 1D-CNN model performance decreased obviously for multilevel residue discrimination. It was found that the multiscale convolution could improve the 1D-CNN model performance. In particular, the asymmetric multiscale convolution had the highest accuracy while keeping a short modeling time. In addition, the model showed a better capability to discriminate beta-cypermethrin than lambda-cyhalothrin. The asymmetric multiscale 1D-CNN model could not accurately differentiate the medium and high contents of lambda-cyhalothrin residues. In addition, the model discrimination accuracy of four-level discrimination was higher than that of three-level discrimination. The overall studies indicated that the model architecture is an important factor affecting its performance. The increase in classification complexity could reduce the model accuracy. But this may also be related to the statistical characteristics of the data. It needs further study.

In general, 1D-CNN algorithms were effective as a spectral analysis strategy. The Vis/NIR spectroscopy combined with multiscale convolution appeared promising for discriminating pesticide residue levels in fruits. In the future, we will collect more samples to eliminate the effect of individual differences and enhance model versatility, and the portable detection devices of pesticide residues will also be focus.

## Figures and Tables

**Table 1:** Hyperparameters used in network layers.

1D-CNN Model	Layer		Hyperparameters				
			Filter Number	Filter Size	Stride	Padding	Activation
Single depth	Conv1		16	1 × 1	2	same	ReLU
	Conv2			3 × 1			
	Conv3			5 × 1			
	Pooling		—	2 × 1			—
Symmetric multiscale	Conv1	C11	16	1 × 1	2	same	ReLU
		C12		1 × 1			
		P13	—	2 × 1			—
	Conv2	C21	16	3 × 1			ReLU
		C22		5 × 1			
		C23		1 × 1			
Asymmetric multiscale	Conv1	C11	16	1 × 1	2	same	ReLU
		C12		1 × 1			
		P13	—	2 × 1			—
		C14	16	1 × 1			ReLU
	Conv2	C21		3 × 1			
		C22		3 × 1			
		C23		1 × 1			
	Conv3	P31	—	5 × 1			—
		C32	16	5 × 1			ReLU
	Conv4			7 × 1			

**Table 2:** An illustration of normalized confusion matrix in two-class discrimination.

Normalized Confusion Matrix		Predicted Class	
		Positive	Negative
Actual Class	Positive	TPR	FNR
	Negative	FPR	TNR

**Table 3:** Spectral data distribution in two-level discrimination of pesticide residues.

Pesticide	Residue Level	Residue Content/(μg·mL^−1^)				Spectral Data
		Range	Max	Min	Mean	
Lambda-Cyhalothrin	1	≤8.50	8.38	0.96	3.79	191
	2	>8.50	32.36	8.60	17.26	189
Beta-Cypermethrin	1*	≤2.20	2.04	0.37	1.18	191
	2*	>2.20	12.74	2.34	6.19	189

**Table 4:** Spectral data distribution in three-level discrimination of pesticide residues.

Pesticide	Residue Level	Residue Content/(μg·mL^−1^)				Spectral Data
		Range	Max	Min	Mean	
Lambda-Cyhalothrin	1	<5.00	4.96	0.96	2.32	121
	2	5.00~12.50	12.50	5.02	8.24	130
	3	>12.50	32.36	12.81	21.01	129
Beta-Cypermethrin	1*	< 1.56	1.55	0.37	0.84	128
	2*	1.56~3.75	3.72	1.56	2.40	127
	3*	>3.75	12.74	3.77	7.73	125

**Table 5:** Spectral data distribution in four-level discrimination of pesticide residues.

Pesticide	Residue Level	Residue Content/(μg·mL^−1^)				Spectral Data
		Range	Max	Min	Mean	
Lambda-cyhalothrin	1	<3.00	2.73	0.96	0.51	93
	2	3.00~8.50	8.38	3.36	1.37	98
	3	>8.50~14.60	14.57	8.60	1.87	92
	4	>14.60	32.36	14.63	5.62	97
Beta-cypermethrin	1*	<1.10	1.09	0.37	0.20	97
	2*	1.10~2.20	2.04	1.11	0.29	94
	3*	>2.20~5.00	4.91	2.34	0.77	99
	4*	>5.00	12.74	5.71	2.08	90

**Table 6:** Results of different models for the discrimination of pesticide residue levels on the Hami melon surface.

Pesticide	1D-CNN Model	Discrimination Accuracy/%				Average Modeling time/s
		Residues	Two-Level	Three-Level	Four-Level	
Lambda-cyhalothrin	Single depth	99.25	92.63	81.05	81.05	6.0
	Symmetric multiscale	100.00	93.68	84.21	85.26	3.3
	Asymmetric multiscale	100.00	93.68	86.32	87.37	4.0
Beta-cypermethrin	Single depth	99.25	92.63	84.21	89.47	5.8
	Symmetric multiscale	100.00	94.74	86.32	91.58	2.8
	Asymmetric multiscale	100.00	95.79	89.47	93.68	3.5

## Data Availability

The data presented in this study are available on request from the corresponding author.
